# Efficiency of Crude Protein Utilisation in Grazing Dairy Cows: A Case Study Comparing Two Production Systems Differing in Intensification Level in New Zealand

**DOI:** 10.3390/ani10061036

**Published:** 2020-06-15

**Authors:** Martín Correa-Luna, Daniel Donaghy, Peter Kemp, Michael Schutz, Nicolas López-Villalobos

**Affiliations:** 1 School of Agriculture and Environment, Massey University, Private Bag 11-222, Palmerston North 4410, New Zealand; D.J.Donaghy@massey.ac.nz (D.D.); P.Kemp@massey.ac.nz (P.K.); N.Lopez-Villalobos@massey.ac.nz (N.L.-V.); 2 Department of Animal Science, University of Minnesota, St. Paul, MN 55108, USA; mschutz@umn.edu

**Keywords:** dietary crude protein utilisation efficiency, dairy cows, grazing, excreta, intensification

## Abstract

**Simple Summary:**

Improving the dietary crude protein utilisation in dairy cows is a key aspect of agronomically and environmentally sustainable production systems. The intensification process of grazing dairy systems identified with the increase of milking cows linked with the addition of supplementary feed along with the increasing use, and particularly inefficient use, of nitrogen fertiliser, has led to increasing pressure on the environment. However, feeding solely on pasture could result in an excess of crude protein intake relative to nutritional requirements, and this could reduce the dietary crude protein utilisation. In this study, we modelled the dietary crude protein utilisation, along with nitrogen excreta partitioning of milking cows, of two contrasting spring-calving pasture-based herds differing in intensification level in New Zealand. We found that feeding diets with higher fresh pasture proportions, such as those employed in low-intensification dairy systems, led to an excess of crude protein intake with greater nitrogen partitioned towards urine, which is sensitive in terms of body water eutrophication. In the high-intensity production system, the inclusion of low-crude protein supplements resulted in better dietary crude protein utilisation, along with less urinary nitrogen losses.

**Abstract:**

In this study, we modelled and compared lactation curves of efficiency of crude protein utilisation (ECPU) and the nitrogen (N) excreta partitioning of milking cows of two contrasting spring-calving pasture-based herds to test some aspects of farming intensification practices on cow performance and N partition. In the low-intensity production system (LIPS), 257 cows were milked once-daily and fed diets comprised of pasture with low supplementary feed inclusion during lactation (304 kg pasture silage/cow). In the high-intensity production system (HIPS), 207 cows were milked twice-daily and fed pasture with higher supplementary feed inclusion (429 kg pasture silage and 1695 kg concentrate/cow). The dietary crude protein (CP) utilisation was calculated for each cow at every herd test date as the ECPU as a proportion of protein yield (PY) from the CP intake (CPI) derived from intake assessments based on metabolisable energy requirements, and the CP balance (CPB) calculated as the difference between CPI and PY. Total N excreta partitioned to faeces (FN) and urine (UN) was estimated by back-calculating UN from FN, considering dietary N, and from N retained in body tissues, taking into account live weight change during the lactation. The higher CPI (2.7 vs. 2.5 kg CP/day), along with the reduced milk yield (1100 kg milk/cow less), of the LIPS cows led to a lower ECPU (23% vs. 31%) and to a higher CPB (2.1 vs. 1.8 kg CP/day) when compared to the HIPS cows. Mean N excreta, and particularly UN, was significantly higher in LIPS cows, and this was explained by higher dietary CP and by the reduced PY when compared to the HIPS cows. Reducing the low-CP supplementation in the “de-intensified” herd lessened the ECPU, resulting in higher UN, which is sensitive in terms of body water eutrophication.

## 1. Introduction

The efficiency with which lactating cows convert dietary crude protein (CP) into milk protein can be measured by different criteria. For instance, it can be measured as the efficiency of CP utilisation (ECPU) calculated as the protein in milk (PY) in a day divided by the daily CP intake (CPI), or calculated considering an approximation of CP balance (CPB) estimated as CPI minus PY [1]. Compared to diets formulated from total mixed rations, diets comprised of predominantly fresh grazed temperate pastures are high in CP [nitrogen (N) concentration × 6.25] concentration in early spring and late autumn, containing mainly rumen-degradable protein at levels that regularly exceed animal requirements [2,3]. Therefore, lower efficiency of N utilisation, with values ranging from 13% to 33%, would be expected in grazing conditions, and this would result in increases in excreted N, predominantly in urine [4], which elevates the nitrate-N levels in soil solution and groundwater [5,6]. Ledgard et al. [5] concluded that N leaching from grazed pastures increased exponentially with increased N inputs and that urinary-N contributed from 70% to 90% of total N leaching. Within grazing conditions, manipulating the dietary CP is limited, but when low N energy-dense concentrate is fed to pastured dairy cows, it was proven as a strategy to provide more energy for microbes to increase the microbial protein synthesis. A study conducted by Mulligan et al. [7] tested the effect of supplementing pastured dairy cows with low vs. high protein concentrate and demonstrated that low protein supplementation increased milk yield and reduced the CPI, and this was observed in improvements in the ECPU. By including low CP concentrates, minor changes in milk protein composition were documented [8], along with an increase of N excreted in faeces rather than urine [7,9].

The excess of dietary CP is rapidly converted to urea to avoid harm from the excess of ammonia. Urea is transported from the plasma and subsequently is transported to other fluids, such as saliva, in order to be recycled or to be excreted in urine, but it can also be found in milk as milk urea nitrogen (MUN). The relationship between MUN and dietary CP was reported to be positive in housed conditions [10,11,12] and in grazing conditions [13,14]. Hence, MUN has been proposed as a proxy to identify excess CP in diets [10]. Since milk is routinely collected and MUN is routinely determined in some countries by milk companies in order to follow animal and dietary characteristics, MUN is proposed as a convenient and non-invasive biomarker for dietary N-use efficiency [10,15] and as a predictor of N excreted through urine into the environment [11,12].

In response to the global demand for dairy products, the New Zealand dairy industry has expanded rapidly since the 1990s [16], with dairy companies processing 7077 million litres of milk in 1990 and 20,702 million litres of milk in 2016 [17]. This expansion of the dairy industry was achieved through, among other factors, the genetic improvement of animals by implementing a well-defined national breeding program, by increasing the amount of bought-in supplementary feeds, and by increasing the use of N fertiliser, with a concomitant increase in the number of cows per ha [16]. Some aspects of this intensification process have reduced the farm N utilisation efficiency and increased the nutrient losses from these systems [18,19]. This has led to increasing pressure on soil and water resources, endangering native vegetation reserves and wetlands, and jeopardising the long-term sustainability of these intensified production systems [20,21]. As more inputs are included in these systems, production costs are likely to increase. In order to maintain profitability, cows would need to be milked at least twice-daily (TAD) to increase the number of secretory cells and promote their activity, which is linked with milk yield at peak lactation and with the persistency of lactation, respectively [22].

Despite the well-documented yield loss of systems with cows milked once-daily (OAD) [23,24,25], some farmers are adopting milking cows OAD throughout a full lactation as a production system for better lifestyle [26] and to reduce production costs [24]. An OAD production system is typically associated with lower intensification, especially lower use of supplementary feeds, and reduced labour [27]. Although previous research analysing OAD production systems focused on animal performance and economic outcomes [23,24,25], there is little information regarding the environmental implications of OAD production systems, especially in terms of ECPU and N losses at both cow and whole-farm levels. Since July 2013, Massey University Dairy 1 farm reduced the stocking rate from 2.7 to 2.1 cows/ha and is managing a low-intensity production system, given the free-draining characteristic of its soils and the proximity to the Manawatu River and to Palmerston North city, to be in line with regional environmental guidelines to reduce nitrate leaching. Cows are milked OAD throughout the lactation with restricted supplementary feed as a strategy to reduce production costs. At the same time, Massey University Dairy 4 farm, which is located on a rural area and is comprised of silt loam soils of less nutrient leaching risk when compared to Dairy 1 farm, is managed as a high-intensity production system with cows milked TAD throughout the season and higher supplementation level included throughout the year. This represented an excellent opportunity to model and compare the dietary crude protein utilisation efficiency and N partitioning of cows of these two research herds throughout an entire lactation. It was hypothesised that the reduction in milk production along with a higher dietary CP in the “de-intensified” herd, as more pasture is fed in combination with less low-CP supplements offered, would decrease the ECPU and increase the N losses. Under these productive management conditions, cows are expected to have a positive energy balance and there might be a link with the CPB. Thus, the objective of this study was to model and compare two measures of dietary CP use efficiency, along with the N excreta partitioning of these two herds of contrasting spring-calving pasture-based production systems differing in intensification level, over an entire lactation in 2016. In addition, the relationship of MUN, along with dietary CP utilisation and with the partitioning of N to excreta, was explored.

## 2. Materials and Methods

### 2.1. Description of Production Systems

#### 2.1.1. Low-Intensity Production System

The Massey University Dairy 1 farm is located on the outskirts of Palmerston North, which is in the lower North Island of New Zealand. This farm is managed as a low-intensity production system (LIPS), with 257 cows milked once-daily throughout the season, with a low stocking rate (2.1 cows/ha). The feed strategy includes fresh ryegrass (*Lolium perenne*)/white clover (*Trifolium repens*) pasture as the main diet component, with restricted supplementation and the sporadic use of grazing crops utilised in summer. The herd consisted of 66 Holstein-Friesian (F), 55 Jersey (J), and 136 Holstein-Friesian/Jersey crossbred cows (F × J) with 12, 15, and 15 primiparous cows, respectively. Calving commenced on 11 July and continued up to 3 October 2016. Cows were milked daily at 06:30 throughout the season. After calving, milking cows had daily access as a single group to a new strip of pasture after each milking and were contained in their allocated forage area through the use of temporary electric fences. From December to March and in April, cows had daily access to a perennial herb-mix crop [mixture of plantain (*Plantago lanceolata*), chicory (*Cichorium intybus*), and red clover (*Trifolium pratense*)] at an allowance of 3.5 kg dry matter (DM) per cow per day. In March and May, alfalfa (*Medicago sativa*) was grazed at an allowance of 3 kg DM per cow per day. Turnips (*Brassica campestris* ssp. *rapifera*) were grazed at an allowance of 2.6 kg DM per cow per day only in February. Pasture silage was fed directly on the paddock in August and from March to May at a rate of 3.5 kg DM per cow per day.

#### 2.1.2. High-Intensity Production System

The Massey University Dairy 4 farm, located adjacent to the Dairy 1 farm, is a farm managed as a high-intensity production system (HIPS), with 207 cows milked twice-daily throughout the season, with a higher stocking rate (2.8 cows/ha). Ryegrass-white clover pasture is also the main feed source but, in this case, higher supplementation level is included throughout the year. The herd was comprised of 51 F and 156 F × J cows, with 5 and 9 primiparous cows, respectively. Calving commenced on 1 July and continued up to 26 September. Cows were milked daily at 05:30 and 14:30 throughout the season. After calving, milking cows had daily access as a herd to a new strip of pasture after each milking. During the lactation, maize (*Zea mays*) silage and grain-based concentrate were fed during the lactation at a rate of 3.5 kg DM per cow per day before the afternoon milking; 2 kg DM per cow per day was fed inside the parlour. Pasture silage was fed directly on the paddock in January at a rate of 3 kg DM per cow per day. In March, dried distillers grains were fed at a rate of 0.75 kg DM per cow per day during the morning milking; turnips were grazed in strips at an allowance of 2 kg DM per cow per day.

### 2.2. Feed Allocation and Chemical Composition Measurements

In the period of 24 h before each monthly herd test, pasture and crops allocated to each herd were sampled in order to quantify the ingredient proportions and to measure the chemical composition. Estimated pasture eaten [kg dry matter (DM) per cow per day] was calculated from pasture disappearance by measuring pregrazing DM minus postgrazing DM on the grazing area assigned per day divided by the total number of cows. The grazing area was measured using a global positioning system. Pre- and postgrazing pasture heights were measured with a rising-plate meter (Jenquip, New Zealand) following a “W” pattern across the grazing area on the basis of 3 sets of 50 readings on each occasion. Pasture mass was subsequently estimated using the following New Zealand national calibration equation for perennial ryegrass-white clover (kg DM/ha = 140 × compressed height + 500) [28]. Estimated crop (herb-mix, lucerne, turnips) consumption was calculated as crop cover pregrazing minus crop cover postgrazing, measured by harvesting three 0.1 m^2^ quadrats to ground level within the area allocated to each herd on a daily basis. Those measurements enabled the calculation of apparent pasture and crop utilisation and also the proportion of feed ingredients allocated to cows in each herd test.

Fresh pasture and crop samples (approximately 1500 g of wet weight) were harvested using the hand-plucking method [29] from about 50 sites on each walking transect to mimic herbage grazed by cows. Samples of maize and pasture silage were taken from the bunker, and grain-based concentrates and dried distillers grains were sampled directly from the feeders while cows were being milked. All samples were collected in a period of 24 hours prior to the milk sampling at 09:00 a.m. All samples were freeze-dried and ground (Wiley mill; Arthur H. Thomas, Philadelphia, PA) to pass through a 1.0-mm screen. The levels of ash, CP, lipid, neutral detergent fibre (NDF), acid detergent fibre, organic matter digestibility, metabolisable energy (ME), and starch and soluble sugars were estimated by near-infrared reflectance spectrometry (NIRS) [30]. Calibrations for each component had been previously developed (Massey University Nutrition Laboratory, Palmerston North, New Zealand) using NIRS after scanning finely-ground pasture samples in the range of 400 to 2500 nm. A Bruker MPA NIRS (Ettlingen, Germany) was used to scan the samples, and the resulting NIRS spectra were analysed using Optic user software version 5.0. (Ettlingen, Germany). The resulting NIRS calibration typically had a correlation of 0.90 when compared to the wet chemistry results for each component. Based on the proportions of each forage allowed and each individual feed ME content, the dietary ME content (megajoules of ME per kg DM) was calculated. Table 1 resumes the annual feed allocation (diet composition) of each production system with its chemical composition.

### 2.3. Animal Measurements

Cows were identified using a radio frequency electronic identification system (Allflex New Zealand Ltd., Palmerston North, New Zealand), enabling daily body weight (BW) and daily BW change measurements to be generated using an automatic race walkover scale (WoW xR-3000, Tru-Test Ltd., Auckland, New Zealand). Milk yield (MY) was determined on each monthly herd test throughout the lactation by using mechanical milk meters (Tru-Test Field Collection meter WB HI/Pullout) provided by Livestock Improvement Corporation (Hamilton, New Zealand). Percentages of fat (FP) and protein (PP) were determined in each herd test using a FossomaticTM MilkoScan FT 120 instrument (Foss Electric, Hillerød, Denmark) on composite afternoon and morning aliquots for HIPS and from a unique sample for LIPS on each sampling date. Somatic cell count was determined using a FossomaticTM FC instrument (Foss Electric, Hillerød, Denmark) and converted to somatic cell score (SCS) as SCS = log2 (somatic cell count). Yields of fat (FY) and protein (PY) were calculated by using daily MY, along with FP and PP, respectively. In early (September), mid (December), and late (March) lactation stages, MUN (mg/dL) content and lactose percentage was determined using a CombiFoss™ 7 instrument (Foss Electric, Hillerød, Denmark), following the CombiFoss technique [31] by MilkTestNZ (Hamilton, New Zealand). Each MUN record was converted into MUN yield (MUNY) (g MUN/cow/day) using daily MY. In synchrony with each herd test, BCS was assigned to each cow by a single research technician using a 10-point scale [32].

Macciotta et al. [33] identified that the mathematical properties of the Legendre polynomials functions were able to recognise a large number of curve shapes, enabling the modeling of the lactation curves of large groups of animals in contrasting productive conditions. In turn, lactation curves for milk production traits and BW were modelled from day 1 to day 275 of lactation, contemplating the maximum DIM of each cow using random regression models with Legendre polynomials of the third order, with the MIXED procedure of the Statistical Analysis System version 9.4 (SAS Institute Inc., Cary, NC, USA). Predicted values for each day were obtained from the polynomial function for each cow. The polynomial utilised is described in equation as

(1) $$Y_{\mathrm{it}}=\alpha_{i0}P_{i0}{+\alpha }_{i1}P_{i1\;}{+\alpha }_{i2}P_{i2}{+\alpha }_{i3}P_{i3}$$

where Y_it_ represents the level of production of a trait _i_ on day _t_ of the lactation after calving, with α being the regression coefficient to predict each trait mentioned above. The Legendre polynomials’ functions of P_Y_ were calculated as

(2) $$P_{0}\left(t\right)=1,$$

(3) $$P_{1}\left(t\right)=x,$$

(4) $$P_{2}\left(t\right)=\frac{1}{2}{(3x}^{2}\;-\;1),\;\mathrm{and}$$

(5) $$P_{3}\left(t\right)=\frac{1}{2}{(5x}^{3}\;-\;3x),\;\mathrm{where}$$

(6) $$x=-1+2\frac{{(t-t}_{\min})}{{(t}_{\max}{-t}_{\min})}\;,$$

according to Silvestre et al. [34].

Prediction of individual daily values of milk production traits along the lactation were accumulated in order to obtain the total lactation yield per cow for MY, milk solids yield (MSY; FY + PY), FY, PY, MUN, and MUNY.

### 2.4. Energy Requirements and Intake Estimates

Values of net energy requirements for maintenance, pregnancy, production, and daily BW variation were based on the French net energy system, where 1 unité fourragère lait is the net energy requirement for lactation equivalent to 1 kg of standard air-dried barley [35], equivalent to 7.11 MJ of net energy or 11.85 MJ of ME. Net energy requirements were calculated using the following equation, with modifications by Berry et al. [36] for dairy cows in grazing conditions:

(7) NEreq=NEm+NEl+NEg+NEp

where NEreq is daily net energy requirement for each cow on each herd test date; NEm is daily net energy requirement for maintenance, including activity calculated as (1.4 + 0.6 × BW/100) × activity allowance factor of 1.2 for grazing conditions; NEl is the net energy for milk production calculated as 0.054 × (FY/100) + 0.031 × (PY/100)+ 0.028 × (LY/100) − 0.015) × MY; NEg accounts for daily body weight variation, assuming an addition of 3.5 units when BW change is positive and −4.5 units when BW change is negative; NEp is daily net energy requirements for pregnancy where unité fourragère lait requirements for the 6th, 7th, and 8th month of pregnancy are 0.9, 1.6, and 2.6, respectively. Total net energy requirements were transformed to ME requirements by multiplying NEreq times 11.85 MJ of ME. Then, apparent DMI (kg DM/cow per day) for all herd test occurrences was estimated by dividing total ME requirements by the dietary ME content in each instance.

### 2.5. Definitions of Nitrogen Utilisation Efficiency and Nitrogen Excreta Estimates

Two different assessments of N utilisation efficiency were used in this study. The ECPU was calculated as a proportion of CPI and from records of PY obtained from the monthly herd tests as

(8) $$\mathrm{ECPU}=\frac{\mathrm{PY}}{\mathrm{CPI}}\;\times \;100$$

with CPI determined by multiplying DMI by the CP concentration in the diet.

A complete N balance is required to examine the partition of total N intake towards N in faeces, urine, milk, and N retained in body tissues and the foetus [37], but these types of studies are expensive and cannot be undertaken on a large number of cows in grazing conditions. However, CPB can be calculated on a daily basis as the difference between CPI and PY [1], where the most inefficient cow has the highest CPB. It should be noted that the CPB cannot replace a full balance study, and this limits the interpretation of the results.

All the excess of dietary CP in the cow would be excreted except for a minor portion that would be retained by cows with positive energy balance. Each kg of BW change was assumed to contain 160 g of CP [38]. A back-calculation for N excreta estimation was undertaken, considering the retention of N in body tissues as constant. Total N excreta comprises N contained in faeces and urine during a given period. Compared to N excreted in urine (UN), N excreted in faeces (FN) is constant relative to DMI in lactating cows [39]. Faeces are the main fate of undigested feed N, undigested microbial N, and endogenous N [6]. Considering this, FN (g N excreted per day) was estimated, employing the formula of Reed et al. [40]:

(9) FN=72.7 − 11.8 × ME − 0.4 × NDF+3.5 × CP+0.2 × ForR+9.3 × DMI − 0.1 × DIM

where ME is the metabolisable energy feed content, NDF is the neutral detergent fibre feed concentration, CP is the crude protein feed concentration, ForR is the proportion of pasture in the diet, DMI is the daily feed intake on dry-matter basis, and DIM is the days in milk. Subsequently, UN (g N urine excreted per day) was estimated as

(10) UN=feed N − FN − milk N − N retention

where UN is the N excreted in urine (g of N per day), feed N represents the intake of N (g of CP intake per day divided by 6.25), milk N (g of N in milk per day), and N retention corresponds to N retained in body tissues according to BW variation (g N retained per day). Similar to the two N use efficiencies analysed in this study (ECPU and CPB), the partition of N excreta was not predicted following the Legendre polynomials’ functions but estimated according to cow performance and dietary characteristics in each case. Measurements of CPI, PY, and CP retained were converted into N by dividing each variable by 6.38 beforehand (as the average N content of protein is 160 g per kg of BW) in order to account for the N partition fractions.

### 2.6. Statistical Analyses

The approach of this study is to examine how the two measures of dietary CP use efficiency and the N partition of pastured dairy cows is altered on different management practices (farm production system and supplementary feed inclusion) considering that these are representative situations of commercial farms with contrasting intensification levels in New Zealand conditions. The estimates of the third-order Legendre polynomial regression coefficients for each cow for each of the lactation measures of yields of milk, fat, protein, lactose, SCS, MUN, MUNY, BW, and BCS, along with lactation length, DMI, CPI, ECPU, and CPB, were analysed using the MIXED procedure of SAS (version 9.4; SAS Institute Inc., Cary, NC). The model included the fixed effects of the farm production system, lactation number, interactions between lactation number and farm production system, and as covariates, the deviation from median calving date, proportion of F, the heterosis effect among F and J, and the random effect of cows to account for repeated measures on the same cow. Estimates of Pearson correlation coefficients and standard errors for actual and predicted values of daily milk, fat and protein yield, somatic cell score, MUN, MUNY, BW, and BCS were obtained with the CORR procedure of SAS.

## 3. Results

Descriptive statistics of herd test records containing yields of milk, fat, lactose, and protein, along with SCS, MUN, and MUNY, are in Table 2. Mean MY of LIPS was 26% lower than HIPS, but that gap was reduced to 15% and 20%, respectively, when comparing FY and PY between the two herds. There were higher mean, minimum, and maximum MUN concentrations in LIPS cows (Table 2 and Figure 2c), and standard deviations for MUN almost doubled in LIPS cows, reflecting larger variations between cows and during the lactation. Results for MUNY were similar among herds.

Irrespective of the group of cows of this study, Pearson linear correlations between predicted and actual values for all traits examined were near unity (Table 3), which depicts the suitability of the Legendre orthogonal polynomials to model the cow performance during lactation. The accuracy of this methodology to model the lactation curves was superior for the milk production traits, with correlations greater than 0.923 when compared to other cow performance parameters such as BW or BCS, with values of 0.988 and 0.868, respectively. Correlations of actual MUN and MUNY with their corresponding predictive values were equal to 0.991 and 0.960 in LIPS, and to 0.968 and 0.898 in HIPS, respectively.

Table 4 has the least squares means of the regression coefficient estimates employed to obtain predicted values for the MY traits and cow performance throughout the lactation. Differences in alphas (*p* < 0.001) are consistent with milk production performance depicted in the descriptive statistics shown in Table 2, with higher α_0_ (intercept) in all traits of milk production and lower α_0_ for SCS in HIPS. A similar trend was observed and confirmed with the descriptive statistics with regards to BW and BCS in both herds. Likewise, α_0_ of MUN was higher in the LIPS cows, corroborating results previously described.

**Figure 1:**
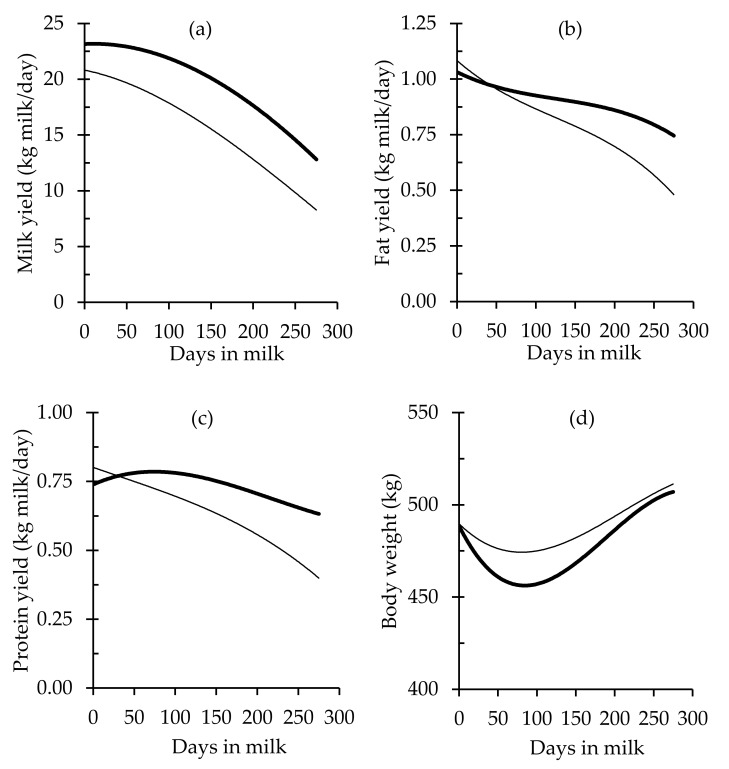
Predicted lactation curves of yields of (**a**) milk, (**b**) fat, and (**c**) protein, and (**d**) body weight of grazing cows throughout a full lactation in two contrasting pasture-based dairy production systems: a low-intensity production system (—) and a high-intensity production system (**—**).

**Figure 2:**
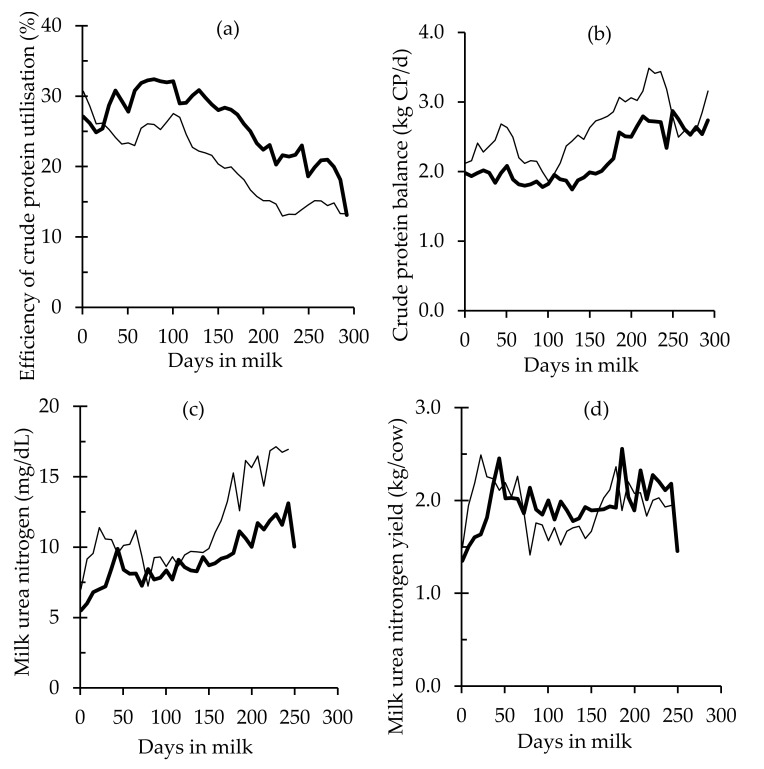
Predicted lactation curves of nitrogen use efficiency calculated as (a) the efficiency of crude protein (CP) utilisation, (b) the CP balance, (c) milk urea nitrogen (MUN; mg/dL), and (d) MUN yield (g) of grazing cows throughout a full lactation in two contrasting pasture-based dairy production systems: a low-intensity production system (—) and a high-intensity production system (—).

There was a significant effect of production system management on total milk production in the present study, with considerably higher MY in HIPS (Figure 1 and Table 5). After adjusting cow performance for heterosis effects between F and J, proportion of F, lactation number, and deviation of median calving date. LIPS cows had 13% lower MSY during the lactation, and this was associated with lower DMI (*p* < 0.001). In the LIPS herd, the higher CPI (<0.001), along with a significant lower PY, resulted in an inferior ECPU (Figure 2a). Compared to the HIPS herd, values for CPB were significantly higher in LIPS (Table 5 and Figure 2b), demonstrating an excess of CP for this group of cows. Values for MUN were significantly lower in HIPS, but MUNY was not different between LIPS and HIPS cows (*p* = 0.058) (Table 5 and Figure 2c,d).

**Figure 3:**
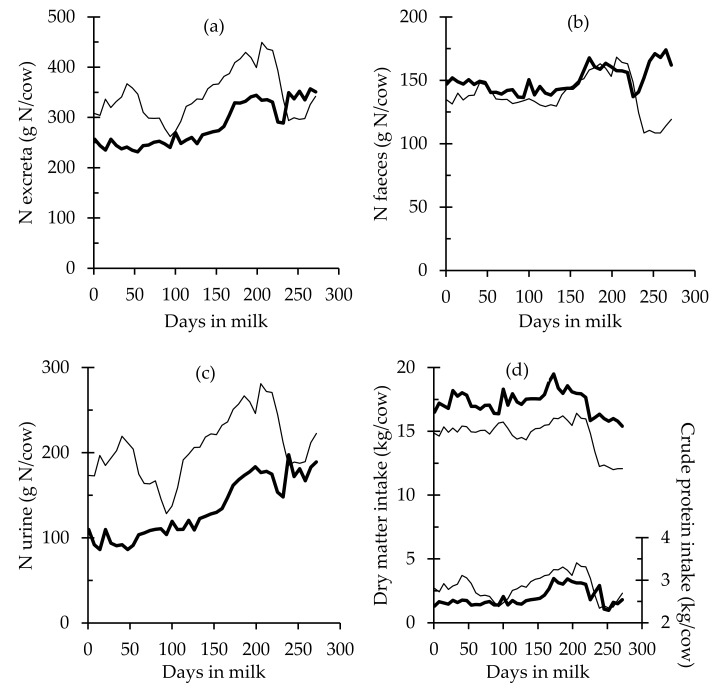
Estimations of (a) total nitrogen (N) excreta per cow (g N/day) and partitioned into (b) urine (g N urine/day) and (c) faeces (g N faeces/day), along with (d) dry matter intake (DMI) per cow (kg DMI/day) and crude protein intake (CPI) per cow (kg CPI/day), in two grazing dairy systems: a low-intensity production system (—) and a high-intensity production system (—).

Estimates of mean N excreta during the lactation (g N per day) was higher in the LIPS cows when compared to the HIPS cows (*p* < 0.001; Figure 3), with a higher proportion of UN (*p* < 0.001; Figure 3b). Mean FN per day was higher in HIPS (*p* = 0.0018; Figure 3c). Cows in HIPS had significantly higher DMI, with a higher proportion of nonpasture supplements of lower CP (compared to fresh grazed pasture) included throughout the lactation, which led to a diet lower in CP (Figure 3d).

## 4. Discussion

In the present study, two measures of dietary CP use efficiency, along with estimations of N excreta partitioning, in two contrasting dairy production systems of dissimilar intensification levels are described. Although it was impractical to allocate two levels of supplementary feed inclusion in each herd milked once or twice daily, no research describing the performance of dietary CP use efficiency during the lactation of pastured cows with varying milking frequency has been found in previous literature. There was a substantial difference in the intercept between regressions of ECPU of LIPS and HIPS herds (Figure 2a) due to differences in milk production and in CPI (Figure 3d). Diets high in CP involve an extra nutritional “cost” to eliminate the surplus N from the animal. A review of N metabolism from a large compiling of studies indicated that 250 g additional ammonia absorption per day as a result of feeding CP above requirements would require an extra 7 MJ per day [41], reducing energy destined for milk production and reducing ECPU. The better BCS of the LIPS cows demonstrated that the higher CPI did not affect the energy balance, and this was due to the lower MY observed in this group of cows. A higher CPB was observed throughout the lactation in the LIPS cows compared to HIPS cows, reflecting more N than could be allocated towards PY (by limiting MY), resulting in an increase in N concentration in excreta. Determination of N losses in skin, scurf, hair, and excreta in the lactating cow is difficult and can lead to sources of errors when studying the kinetics of N [37]. Nevertheless, the substantial gap in ECPU (and CPB) between LIPS and HIPS is undeniable and overcomes such potential measurement errors (Figure 2a,b).

Contrary to the intercepts for ECPU regressions, the intercepts for CPB tended to be similar for both herds (Figure 2b). This is due to a counterbalance between the differences in CPI and PY of both herds in early lactation. The negative slope in CPB of both LIPS and HIPS herds demonstrated that the body tissue mobilisation in early lactation contributed not only with energy but also with protein towards the intense metabolic demand that a transition cow (from dry period to peak lactation) faces in its first days of lactation [42]. By feeding diets with higher supplementation and milking cows twice-daily, HIPS cows produced more milk, and this signified that more CP was partitioned towards PY and this was seen in less CPB when compared to LIPS cows. Attention should be paid to management practices that reduce milk production because all the CP not allocated in milk would escape in excreta.

The liver, along with the portal-drained viscera, are tissues of high internal N metabolic activity, and while they represent less than 10% of BW, they are responsible for the major N metabolic exchange in ruminants [41], including ureagenesis, a vital mechanism to overcome poisoning from the excess of ammonia present in the systemic circulation. That labile N pool is highly influenced by feeding management [43], among other factors. In turn, urea is transported from the plasma to other fluids such as saliva in order to be recycled and is excreted mainly in urine, but it is also present in milk. The relationship between urine urea N and MUN was recognised by a number of authors [11,12]. In contrast to the higher MUN in the LIPS herd when compared to the HIPS herd in the present study, earlier studies of Friggens and Rasmussen [44] and Nielsen et al. [45] reported increases in MUN with decreases in milking intervals, but the explanations were speculative, with authors suggesting that there might be an interaction with time of feed intake and time of milk sampling that results in disturbances in MUN determinations. Irrespective of management practices in each herd, the results of the present study confirmed a positive relationship between MUN and CPI [46,47]. Compared to the HIPS herd, CPI of the LIPS herd was always higher and was accompanied by higher MUN throughout the lactation (Figure 2c), with the exception of the period around day 90 of lactation when values of CPI of the HIPS herd were similar to the LIPS herd, resulting in similar MUN. Cows milked in the LIPS herd were fed mainly ryegrass pastures of high quality and high CP concentration, with minor inclusion of supplements in the diet. This represents an imbalance of energy: the protein ratio [2] leading to increases in N excreta and MUN. On the contrary, De Campeneere et al. [48] reported higher urine N levels along with lower plasma urea N and MUN for cows fed pasture silage compared to cows fed maize silage diets and attributed this to the high consumption of potassium and sodium from pasture silage diets. However, there might be a confounding factor, considering the differences in DMI between the pastured-dairy cows in this present study and the cows in housed production systems utilised by De Campeneere et al. [48]. The ensiling process can considerably alter the nutritive value of forage, leading to increases in nonprotein N at the expense of true protein, the rate of proteolysis, and concentrations of volatile fatty acids, as well as reductions in carbohydrate content [49]. While finding substantial differences in ECPU between these two herds, MUNY was similar for both herds (Table 5). This equity was in response to higher MY, along with lower MUN in the HIPS herd and the opposite in the LIPS herd. Because of this compensation between MUN and MY, an erratic relationship of MUN with both ECPU and CPB is reported in this study. At day 90 of lactation, cows had the same MUN and equal CPI, but the ECPU was higher and CPB was lower in the HIPS cows. Similarly, Barros et al. [46] reported some inconsistencies in the relationship of ECPU and MUN, with a positive relationship between MUN and feed efficiency of 0.71, leading to higher MY and PY. On the contrary, Nousiainen et al. [12] improved the estimates of UN excretion and ECPU with back-calculations by including MUNY. However, Nousiainen et al. [12] used housed cows fed total mixed rations with lower dietary CP compared to the grazing conditions of the present study. Several authors and results from the present study suggest that the relationship between MUN and ECPU is governed by several factors, including MY, BW, lactation stage, between-animal variation, and nutritional management [38,43].

This study indicated that irrespective of the lower DMI of the LIPS cows, CPI was increased through ingestion of a larger proportion of fresh pasture with higher digestible N. Compared to the HIPS cows, the greater CPI along with lower PY of the LIPS cows (Table 5 and Figure 3d) resulted in a lower ECPU and a greater CPB with inevitable increases in N excreta. Moreover, in the period around day 90 of lactation, similar CPI for cows of both herds resulted in higher UN in LIPS cows, and this was confirmed by the disparity in the ECPU and in the CPB analysed in this study at this particular time. A number of authors also reported the same strong negative relationship between CP in the diet and the CP use efficiency, and with the increase in N excreta of lactating cows [9,46,47].

Results from the present study confirmed on a daily basis that feeding concentrates to supplement grass-based diets could shift N excretion from urine to faeces [7,9]. While LIPS cows had mean daily calculated UN values of 199 g N per day compared to 134 g N per day in HIPS cows, HIPS cows had 139 g N per day in FN compared to 134 g N per day in LIPS cows. Proportionally, cows belonging to HIPS partitioned more N towards faeces, in which N is less volatile than N in urine, which may be converted to ammonia and nitrous oxide at a slower rate [5,19]. This aspect of the alteration in N excreta partition towards faeces could be interesting from an environmental perspective, considering that reducing the rate of N loss may result in less N leached from the system [5].

In the first 100 days of lactation, HIPS cows had greater mobilisation of body tissue (Figure 1d), which contributed to the CP requirements of those cows (considering that 1 kg of BW mobilisation equates to 160 g of CP; [38]) facing a high demand for nutrients for milk production and other metabolic processes, including the uterine involution postpartum. If not for this mobilisation of BW, it would have been difficult for HIPS cows to achieve the recorded high MY (mean peak milk production of 25 kg) with a diet comprising 14.5% CP in early and mid-lactation, reducing the ECPU by achieving lower PY. Trevaskis and Fulkerson [50] suggested that the lower level of MUN aligned with peak milk yield was attributed to nutrient mobilisation in early lactation, as more N available would be potentially allocated to milk protein synthesis, affecting the ECPU and the CPB, as it was observed in this study. A recent study by Daniel et al. [42] recommended subdividing the lactation into stages when analysing protein and energy requirements of lactation.

Additional mitigation techniques must be studied in order to alleviate the N loss to the environment of grass-based systems, specifically in low-intensity OAD systems. For example, the present study indicates that feeding low CP concentrates to dairy cows grazing good-quality pasture would reduce their CPI, with no negative effect on feed intake or milk production, and at the same time, some shifting of N excreta from urine towards faeces can be achieved. Moreover, the implementation of strategic supplementation in low intensity grazing systems should need to be carefully designed by detecting true deficits and surplus of nutrients (primarily energy and protein) as a promising way for dairy farmers to be more competitive, while reducing the environmental footprint [15,18,19].

## 5. Conclusions

Both measures of CP use efficiency in LIPS and in HIPS were driven by the CPI and by the levels of milk production. The combined effect of extra supplementary feed and milking frequency employed, considering each intensification approach in each production system, affected the overall milk production performance, and, consequently, the ECPU was higher in HIPS cows. Moreover, the ECPU of LIPS cows was reduced by feeding diets comprising mainly fresh pasture high in CP. The LIPS cows had a higher CPB as a result of lower milk protein synthesis and higher dietary CP when compared to HIPS cows.

Lactation curves of MUN for LIPS and HIPS cows were in line with levels of CP fed, but no association was found with CP use efficiency using the approaches proposed in the present study. The compensation observed between MY and MUN levels in both herds resulted in similar MUNY, and this explained the similarities in MUN levels at some stages of the lactation. Thus, an erratic relationship of MUN with both ECPU and CPB was observed in this study.

Due to an excess of dietary CP in LIPS cows, UN was elevated, and as this is the most sensitive fraction of excreta. Mitigating strategies must be studied and evaluated in regards to alleviating N loss to the environment in lower-input dairy production systems. It is noteworthy to highlight that this paper demonstrated that some management practices considered as part of the deintensification of pasture-based dairy production systems, such as the reduction of supplementary feed, are worthy of consideration since a detrimental effect on the efficiency of CP utilisation with more N partitioned towards urine was observed.

## Figures and Tables

**Table 1:** Mean feed allocation and chemical composition offered before each herd test in two contrasting pasture-based dairy production systems. Chemical composition expressed in metabolisable energy (ME, MJ ME/kg DM), and in percentages of crude protein (CP), acid detergent fibre (ADF), neutral detergent fibre (NDF), and organic matter digestibility (OMD).

Item	Production System												
	Low Intensity						High Intensity						
	Pasture ^1^	Pasture Silage	Brassica	Herb-Mix ^2^	Lucerne	Total	Pasture ^1^	Pasture Silage	Brassica	Maize Silage	Concentrate ^3^	DDG ^4^	Total
Feed allocation, % DM	92	3	1	3	1	100	60	8	1	15	15	1	100
ME, MJ ME/kg DM	11.0	10.7	11.7	12.1	9.9	11.1	10.5	10.9	7.8	10.4	12.6	9.5	10.3
CP, % of DM	20	14	16	20	25	19	19	12	22	7	17	21	16
ADF, % of DM	22	34	22	15	21	22.8	24	40	18	28	2	11	21
NDF, % of DM	44	50	34	29	34	38.2	46	56	31	37	23	28	37
OMD, % of DM	75	67	79	83	68	74.4	73	68	81	-	-	-	74

^1^ Perennial ryegrass (*Lolium perenne*)-white clover (*Trifolium repens*) pasture. ^2^ Herb-mix crop comprised of plantain (*Plantago lanceolata*), chicory (*Cichorium intybus*), and red clover (*Trifolium pratense*). ^3^ Grain-based concentrate. ^4^ Dried distillers grains.

**Table 2:** Descriptive statistics of the lactation length records and herd test records of the yield of milk (MY), fat (FY), protein (PY) and lactose yields (LY), somatic cell scores (SCS), milk urea nitrogen (MUN), MUN yield, body weight, and body condition score (BCS) of grazing cows throughout a full lactation in two contrasting pasture-based dairy production systems.

Variable	Production System									
	Low Intensity					High Intensity				
	N	Mean	SD	Min	Max	N	Mean	SD	Min	Max
Lactation length, days	257	269	33	127	319	207	272	36	110	321
Milk yield, kg/day	2284	15.7	6.0	0.9	36.6	1217	21.2	5.5	3.0	37.7
Fat yield, kg/day	2284	0.8	0.3	0.1	2.5	1217	1.0	0.2	0.2	1.5
Protein yield, kg/day	2284	0.6	0.2	0.1	1.3	1217	0.8	0.2	0.2	1.4
Lactose yield, kg/day	726	0.8	0.3	0.1	1.9	558	1.1	0.3	0.5	1.9
Somatic cell score ^1^	2276	5.76	1.59	0.01	12.43	1217	5.13	1.45	1.58	12.02
MUN ^2^, mg/dL day	726	13.11	4.70	4.25	28.83	557	9.72	2.58	2.84	18.42
MUNY ^3^, g/day	726	1.9	0.8	0.3	4.7	557	2.1	0.6	0.4	3.9
Body weight, kg	2359	487	70	320	684	1999	502	62	352	770
Body condition score	2060	4.6	0.4	3.0	6.5	1405	4.2	0.4	3.0	5.5

^1^ The somatic cell count records were log2-transformed to SCS. ^2^ Milk urea nitrogen. ^3^ Milk urea nitrogen yield. Body condition score on a 1–10 scale.

**Table 3:** Estimates of Pearson correlation coefficients and standard error for actual and predicted ^1^ values of daily milk production traits, body weight, and body condition score in two contrasting pasture-based dairy production systems: a low-intensity production system (LIPS) and a high-intensity production system (HIPS).

Trait	Overall	Production System	
		LIPS	HIPS
Milk yield, kg/day	0.997 ± 0.003	0.996 ± 0.004	0.995 ± 0.005
Fat yield, kg/day	0.992 ± 0.004	0.992 ± 0.006	0.990 ± 0.007
Protein yield, kg/day	0.994 ± 0.004	0.995 ± 0.005	0.991 ± 0.007
Somatic cell score ^2^	0.923 ± 0.005	0.922 ± 0.006	0.916 ± 0.008
Milk urea nitrogen, mg/dL day	0.989 ± 0.003	0.991 ± 0.004	0.968 ± 0.008
Milk urea nitrogen yield, g/day	0.941 ± 0.007	0.960 ± 0.007	0.898 ± 0.014
Body weight, kg	0.988 ± 0.002	0.995 ± 0.001	0.976 ± 0.003
Body condition score ^3^	0.868 ± 0.006	0.846 ± 0.009	0.804 ± 0.012

^1^ The predicted values were modelled with a third-order Legendre polynomial. ^2^ The somatic cell count records were log2-transformed to somatic cell score. ^3^ Body condition score on a 1–10 scale.

**Table 4:** Least squares means and standard errors of the estimates of regression coefficients of the lactation curves for milk, fat, protein and lactose yields, somatic cell score, milk urea nitrogen (MUN) and MUN yield, body weight and body condition score modelled with a third-order Legendre polynomial fitted to grazing cows throughout a full lactation in two contrasting pasture-based dairy production systems: a low-intensity production system (LIPS) and a high-intensity production system (HIPS).

Trait	Herd	α_0_	α_1_	α_2_	α_3_
Milk yield, kg/day	LIPS	15.20 ^b^ ± 0.16	−6.94 ^b^ ± 0.12	−1.13 ^b^ ± 0.06	0.21 ^b^ ± 0.02
	HIPS	19.33 ^a^ ± 0.22	−5.83 ^a^ ± 0.17	−1.90 ^a^ ± 0.09	0.10 ^a^ ± 0.02
Fat yield, kg/day	LIPS	0.78 ^b^ ± 0.01	−0.29 ^b^ ± 0.01	−0.03 ^b^ ± 0.00	−0.04 ^b^ ± 0.00
	HIPS	0.89 ^a^ ± 0.01	−0.13 ^a^ ± 0.01	−0.02 ^a^ ± 0.00	−0.03 ^a^ ± 0.00
Protein yield, kg/day	LIPS	0.62 ^b^ ± 0.01	−0.21 ^b^ ± 0.01	−0.04 ± 0.00	−0.01 ^b^ ± 0.00
	HIPS	0.73 ^a^ ± 0.01	−0.08 ^a^ ± 0.01	−0.05 ± 0.00	0.02 ^a^ ± 0.00
Lactose yield, kg/day	LIPS	0.77 ^b^ ± 0.01	−0.37 ± 0.01	0.01 ^b^ ± 0.00	0.05 ^a^ ± 0.00
	HIPS	1.07 ^a^ ± 0.01	−0.39 ± 0.01	0.02 ^a^ ± 0.00	−0.05 ^b^ ± 0.00
Somatic cell score ^1^	LIPS	5.80 ^a^ ± 0.07	1.12 ^a^ ± 0.05	0.04 ^b^ ± 0.04	0.08 ± 0.02
	HIPS	5.02 ^b^ ± 0.10	0.71 ^b^ ± 0.07	0.66 ^a^ ± 0.05	0.03 ± 0.03
Milk urea, mg/dL day	LIPS	14.49 ^a^ ± 0.14	7.64 ^a^ ± 0.14	6.00 ^a^ ± 0.15	−0.59 ± 0.05
	HIPS	10.94 ^b^ ± 0.20	3.91 ^b^ ± 0.19	3.26 ^b^ ± 0.21	−0.49 ± 0.08
Milk urea yield, g/day	LIPS	1.99 ^b^ ± 0.03	−0.17 ± 0.02	0.48 ^b^ ± 0.01	−0.33 ± 0.01
	HIPS	2.10 ^a^ ± 0.04	−0.14 ± 0.03	0.50 ^a^ ± 0.01	−0.33 ± 0.02
Live weight, kg	LIPS	488.06 ^a^ ± 2.71	18.35 ^b^ ± 0.94	13.48 ^b^ ± 0.71	−6.15 ^a^ ± 0.91
	HIPS	477.34 ^b^ ± 3.72	23.81 ^a^ ± 1.29	20.61 ^a^ ± 0.98	−13.66 ^b^ ± 1.25
Body condition score ^2^	LIPS	4.59 ^a^ ± 0.02	−0.13 ± 0.01	0.23 ^b^ ± 0.01	0.05 ^a^ ± 0.00
	HIPS	4.25 ^b^ ± 0.02	−0.12 ± 0.01	0.27 ^a^ ± 0.01	−0.01 ^b^ ± 0.01

^a b^ Means with different superscripts within trait indicates they were significantly different (*p* < 0.05). ^1^ The somatic cell count records were log2-transformed to somatic cell score. ^2^ Body condition score on a 1–10 scale.

**Table 5:** Least squares means (± standard errors) of lactation length, total yield of milk, milk solids, fat, protein and lactose, SCS, body weight and body condition score, dry matter intake (DMI), crude protein (CP) intake (CPI), milk urea nitrogen (MUN) and MUN yield (MUY), efficiency of CP utilisation (ECPU), and CP balance (CPB) of grazing cows throughout a full lactation in two contrasting pasture-based dairy production systems.

Item (per Cow)	Production System		
	Low Intensity	High Intensity	*p* – Value ^1^
N	257	207	
Lactation length, days	272 ± 2	271 ± 3	0.719
Total milk yield, kg	4232.40 ± 55.3	5332.10 ± 75.9	<0.001
Total milk solids yield, kg	387.6 ± 4.4	443.7 ± 6.1	<0.001
Total fat yield, kg	217.4 ± 2.5	243.9 ± 3.5	<0.001
Total protein yield, kg	170.5 ± 2	199.7 ± 2.8	<0.001
Total lactose yield, kg	214.7 ± 3	296.3 ± 4.1	<0.001
Somatic cell score ^2^	5.73 ± 0.07	4.95 ± 0.1	<0.001
Body weight, kg	487 ± 3	475 ± 4	0.012
Body condition score ^3^	4.6 ± 0.02	4.26 ± 0.02	<0.001
DMI, kg/day	16.21 ± 0.07	18.49 ± 0.13	<0.001
CPI, kg/day	3.13 ± 0.01	2.8 ± 0.03	<0.001
MUN, mg/dL	13.20 ± 0.34	9.99 ± 0.23	<0.001
MUNY, g	535.06 ± 19.6	565.01 ± 12.57	0.058
ECPU, %	20.13 ± 0.13	27.65 ± 0.33	<0.001
CPB, kg CP/day	2.51 ± 0.01	2.03 ± 0.03	<0.001

^1^ Differences between treatments were considered significant at *p* < 0.05. ^2^ The somatic cell count records were log2-transformed to somatic cell score. ^3^ Body condition score on a 1–10 scale.
